# Harms, benefits and costs of fecal immunochemical testing versus guaiac fecal occult blood testing for colorectal cancer screening

**DOI:** 10.1371/journal.pone.0172864

**Published:** 2017-03-15

**Authors:** S. Lucas Goede, Linda Rabeneck, Marjolein van Ballegooijen, Ann G. Zauber, Lawrence F. Paszat, Jeffrey S. Hoch, Jean H. E. Yong, Sonja Kroep, Jill Tinmouth, Iris Lansdorp-Vogelaar

**Affiliations:** 1 Department of Public Health, Erasmus University Medical Center, Rotterdam, The Netherlands; 2 Prevention and Cancer Control, Cancer Care Ontario, Toronto, Canada; 3 Institute for Clinical Evaluative Sciences, Toronto, Canada; 4 Department of Medicine, University of Toronto, Toronto, Canada; 5 Department of Epidemiology and Biostatistics, Memorial Sloan Kettering Cancer Center, New York, NY, United States of America; 6 Centre for Excellence in Economic Analysis Research, Li Ka Shing Knowledge Institute, St. Michael's Hospital, Toronto, Canada; 7 Department of Medicine, Division of Gastroenterology, Sunnybrook Health Sciences Centre, Toronto, Canada; Chang Gung Memorial Hospital Kaohsiung Branch, TAIWAN

## Abstract

**Background:**

The ColonCancerCheck screening program for colorectal cancer (CRC) in Ontario, Canada, is considering switching from biennial guaiac fecal occult blood test (gFOBT) screening between age 50–74 years to the more sensitive, but also less specific fecal immunochemical test (FIT). The aim of this study is to estimate whether the additional benefits of FIT screening compared to gFOBT outweigh the additional costs and harms.

**Methods:**

We used microsimulation modeling to estimate quality adjusted life years (QALYs) gained and costs of gFOBT and FIT, compared to no screening, in a cohort of screening participants. We compared strategies with various age ranges, screening intervals, and cut-off levels for FIT. Cost-efficient strategies were determined for various levels of available colonoscopy capacity.

**Results:**

Compared to no screening, biennial gFOBT screening between age 50–74 years provided 20 QALYs at a cost of CAN$200,900 per 1,000 participants, and required 17 colonoscopies per 1,000 participants per year. FIT screening was more effective and less costly. For the same level of colonoscopy requirement, biennial FIT (with a high cut-off level of 200 ng Hb/ml) between age 50–74 years provided 11 extra QALYs gained while saving CAN$333,300 per 1000 participants, compared to gFOBT. Without restrictions in colonoscopy capacity, FIT (with a low cut-off level of 50 ng Hb/ml) every year between age 45–80 years was the most cost-effective strategy providing 27 extra QALYs gained per 1000 participants, while saving CAN$448,300.

**Interpretation:**

Compared to gFOBT screening, switching to FIT at a high cut-off level could increase the health benefits of a CRC screening program without considerably increasing colonoscopy demand.

## Introduction

In most developed countries, including Canada, colorectal cancer (CRC) is the second leading cause of cancer deaths and the third most commonly diagnosed cancer.[1, 2] Screening for CRC and its precursor lesions, adenomas, can detect colorectal neoplasia at an earlier stage when treatment is potentially more effective, resulting in reduced CRC incidence and mortality.[3, 4]

Like a number of regions around the world,[5, 6] the province-wide ColonCancerCheck screening program in Ontario, uses the guaiac fecal occult blood test (gFOBT) to screen individuals at average risk of CRC.[7] Fecal immunochemical testing (FIT) offers several advantages over gFOBT, including greater sensitivity, no need for dietary restrictions and automated processing of test kits.[8] However, depending on the cut-off level used FIT also has a lower specificity, which is associated with increased colonoscopy demand.

At the time of the funding announcement and public launch of the ColonCancerCheck program, the evidence base to support FIT was increasing, but FIT was not yet endorsed by the Canadian Task Force on Preventive Health Care.[9] Hence the implementation of gFOBT by the program. Currently the evidence base has increased sufficiently for the program to consider replacing the gFOBT with FIT as the screening test. In order to inform this decision, the aim of the present study is to compare the costs and benefits of gFOBT and FIT screening in average risk individuals.

## Methods

We used the MISCAN-Colon microsimulation model to estimate the quality adjusted life years (QALYs) gained and costs of gFOBT and FIT screening with varying screening age ranges and intervals, and various FIT cut-off levels in a cohort of average risk Ontarians. Cost-efficient strategies were determined for different levels of available colonoscopy capacity.

### MISCAN-colon microsimulation model

The MISCAN-colon model and the data sources that inform the quantifications of the model are described in detail in S1 Appendix and in previous publications.[10–12] In brief, the MISCAN-colon model simulates the life histories of individuals from birth to death. CRC arises in the population according to the adenoma-carcinoma sequence.[13, 14] More than one adenoma can occur in an individual and each adenoma can independently develop into CRC. Adenomas can progress in size from small (≤5 mm) to medium (6–9 mm) to large (≥10 mm), and some may eventually become malignant. A preclinical (i.e., not detected) cancer has a chance of progressing through stages I to IV and may be detected by diagnostic work-up of symptoms at any stage. After the diagnosis of CRC, survival depends on the stage at diagnosis. At any time during their life individuals may die of other causes.

With screening, an individual with a positive test will be referred for diagnostic colonoscopy for possible removal of adenomas and detection of cancers. In this way CRC incidence and mortality can be reduced. The life years gained (LYG) by screening are calculated as the difference in model-predicted life years lived in the population with and without CRC screening.

The validity of the MISCAN-colon model has been successfully tested on the results of large screening and surveillance studies, such as the randomized trials of gFOBT in Minnesota, Funen, and Nottingham,[12] the CoCap sigmoidoscopy study,[15] and the National Polyp Study.[16] In addition, the model was able to explain observed CRC incidence and mortality trends in the United States when accounting for risk factor trends, screening practice, and chemotherapy.[17] For FIT screening, the simulated stage distribution of screen-detected cancers and the simulated mortality effects were consistent with data from population-based studies.[18, 19] In addition, model-predicted adenoma and cancer detection rates for different cut-off values of FIT showed good concordance with rates observed in Dutch pilot studies (S1 Table).

### Study population

We modeled a cohort of 40-year-old screening participants at average risk of CRC which was followed until death. The CRC incidence and stage distribution were calibrated to incidence data from the Canadian Cancer Registry for 2001, which was prior to the introduction of screening.[20] The model used all-cause mortality estimates from the 2009–2011 Ontario life tables.[21] Because stage-specific data on CRC relative survival were not available for Canada, we assumed similar relative survival as observed in the Surveillance, Epidemiology, and End-Results (SEER) database in the US, in the period 2000–2003.[22]

### Screening strategies

We considered screening strategies for both gFOBT and FIT varying by age of starting screening (40, 45, 50, 55, 60 or 65 years), age of stopping screening (70, 75, 80 or 85 years), screening interval (1, 1.5, 2, or 3 years), and FIT cut-off level used to define a positive test result (50, 75, 100, 150 and 200 ng Hb/ml). The combinations of these variables resulted in 576 unique screening strategies. We used common random numbers for the simulation of every screening strategy to reduce differences in outcomes between strategies due to random variability.

After a positive test result individuals were referred for diagnostic colonoscopy. Depending on the number and size of adenomas detected, the individual would be recommended for surveillance colonoscopy based on current guidelines.[23]

### Test characteristics

The test characteristics of gFOBT were based on a prior calibration of the MISCAN-Colon model to three large gFOBT trials (Table 1).[12] It was assumed that, the probability a CRC bleeds and thus the sensitivity of gFOBT for CRC depends on the time to clinical diagnosis, i.e. cancers that bleed do so increasingly over time, starting in occult fashion and progressing to grossly visible bleeding. The test characteristics of FIT (OC-Sensor Micro; Eiken Chemical Co, Tokyo, Japan) were fitted to the FIT positivity rates and detection rates of adenomas and CRC observed in the first screening round of two Dutch randomized trials.[24–26] We considered FIT cut-off levels of 50, 75, 100, 150 and 200 ng Hb/ml, yielding different combinations of sensitivity and specificity. The test characteristics of colonoscopy were based on a systematic review of polyp miss rates in tandem colonoscopy studies.[27] The lack of specificity of colonoscopy reflects the detection of hyperplastic polyps, which are not explicitly simulated in the MISCAN-Colon model.[28] Additional biopsy costs were assumed for procedures where biopsies were performed and in which, in retrospect, no adenomas were detected.

**Table 1:** Test characteristics of the screening tests used in the model.

Screen test	Specificity (%)	Sensitivity* (%)					
		Adenoma			CRC		
		Small (≤5mm)	Medium (6-9mm)	Large (≥10mm)	Early preclinical†	Late preclinical†	Average
gFOBT	98	2	3	8	20	52	33
FIT 50	96	4	15	37	52	83	65
FIT 75	97	3	9	31	48	81	62
FIT 100	98	2	7	28	43	77	57
FIT 150	98	2	5	25	41	76	56
FIT 200	99	1	4	21	40	76	55
Colonoscopy‡	90	75	85	95	95	95	95

CRC, colorectal cancer; gFOBT, guaiac fecal occult blood test; FIT, fecal immunochemical test.

* Sensitivity is presented per participant for fecal occult blood tests and per lesion for colonoscopy.

† It was assumed that the probability a CRC bleeds and thus the sensitivity of gFOBT and FIT for CRC depend on the time to clinical diagnosis, based on a prior calibration of the MISCAN-Colon model to three gFOBT trials.[12] This result is to be expected when cancers that bleed do so increasingly over time, starting in occult fashion and progressing to grossly visible bleeding.

‡ Colonoscopy was only used during follow-up and surveillance after a positive gFOBT or FIT. The lack of specificity of colonoscopy reflects the detection of hyperplastic polyps, which are not explicitly simulated by the MISCAN-Colon model.[28] Additional biopsy costs were assumed for procedures where biopsies were performed and in which, in retrospect, no adenomas were detected.

### Health-related quality of life

Health benefits were expressed in quality adjusted life years (QALYs) gained. In the model, health-related quality of life declines with increasing age based on a large longitudinal study on the quality of life of Canadians.[29] We incorporated utility losses associated with colonoscopy and its associated complications and CRC using a multiplicative approach (Table 2). Losses in health utility (i.e. loss of quality of life) associated with CRC were based on a recent literature review (Table 2).[30] We assumed a utility loss equivalent to 2 days of life per colonoscopy performed (0.0055 QALYs), and 2 weeks of life for non-lethal complications (0.0384 QALYs).

**Table 2:** Utility weights used in the model.

Variable	Utility loss			
**Screening, per event**				
gFOBT	-			
FIT	-			
Colonoscopy, no polypectomy	0.0055			
Colonoscopy, polypectomy	0.0055			
Complication, bleeding*	0.0384			
Complication, perforation*	0.0384			
**Treatment, per person year of CRC care[30]**†	*Initial care*	*Continuous care*	*Terminal care*, *death CRC*	*Terminal care*, *death other causes*
Stage I	0.15	0.10	0.29	0.10
Stage II	0.15	0.10	0.29	0.10
Stage III	0.15	0.10	0.29	0.10
Stage IV	0.34	0.29	0.29	0.29

gFOBT: guaiac fecal occult blood test; FIT: fecal immunochemical test; CRC: colorectal cancer.

*We assumed a utility loss equivalent to 2 days of life per colonoscopy performed (0.0055 QALYs) and 2 weeks of life for non-lethal complications (0.0384 QALYs). We assumed complications with bleeding in 1.64 per 1,000 procedures, and complications with perforation in 0.85 per 1,000 procedures. In addition, we assumed 1/14,000 colonoscopies resulted in fatal complications.

† CRC treatments were divided into three clinically relevant phases—initial, continuous and terminal care. The initial phase was defined as the first 12 months following diagnosis, the terminal phase was defined as the final 12 months of life, and the continuous phase was defined as all months between the initial and terminal phase. For patients surviving less than 24 months, the final 12 months were allocated to the terminal phase. The remaining months of observation were allocated to the initial phase.

### Costs

The analysis was conducted from a third party health-care payer perspective. All costs were expressed in 2013 Canadian dollars (Table 3). The cost of gFOBT included costs of test kit, dispensing fee, postage, lab processing, communicating results to the participants and collecting data for the screening registry, and was obtained from the ColonCancerCheck program. Since FIT is currently not funded in Ontario, the costs of test kit and processing are unknown. Therefore we estimated the costs of FIT test kit and processing based on the difference between gFOBT and FIT in a Dutch screening trial[31, 32], and applied this difference to the cost of gFOBT in Ontario. We assumed that the dispensing fee and communication of the test results would be identical to gFOBT. The costs attributable to CRC care by CRC stage and phase of care (initial, continuing, and terminal care) included outpatient visits, hospitalizations, treatment, home care, long-term care, and rehabilitation. The costs were estimated using health care administrative data in a matched cohort study, which compared the health care costs of CRC patients with their age- and sex-matched controls (manuscript in preparation).

**Table 3:** Cost estimates used in the model (2013 Canadian dollars).

Variable	Cost (CAN$)				Source
**Fixed program costs per year (assumed identical for gFOBT and FIT screening)**	Year 1: 6,592,000, Year 2: 15,151,000, Year 3: 13,536,000, Year 4: 10,876,000, Year 5: 11,071,000, Year 6+: 10,652,000				ColonCancerCheck program*
**Screening, per event**					
gFOBT	28.23				ColonCancerCheck program*
FIT†	31.11				ColonCancerCheck program*, [31, 32]
GP visit after positive stool test	34.73				[46]
Colonoscopy, no polypectomy	872				[46, 47]
Colonoscopy, polypectomy	1,097				[46, 47]
Complication, bleeding‡	3,521				[45, 48]
Complication, perforation‡	34,412				[45, 48]
**Treatment, per person year of CRC care§**	*Initial care*	*Continuous care*	*Terminal care*, *death CRC*	*Terminal care*, *death other causes*	
Stage I	28,981	7,442	302,484	29,780	Matched cohort study using health care administrative data (manuscript in preparation)
Stage II	43,348	10,435	202,540	37,411	
Stage III	62,259	13,344	134,354	31,334	
Stage IV	83,440	42,551	117,128	29,328	

gFOBT: guaiac fecal occult blood test; FIT: fecal immunochemical test; GP: general practitioner; CRC: colorectal cancer.

* The fixed program costs include costs for the screening registry, program infrastructure, communications and advertising, and sending activity reports to primary care physicians. Personal communication with co-author Dr. Linda Rabeneck, Vice President Prevention and Cancer Control at Cancer Care Ontario.

† FIT is currently not funded in Ontario, therefore the costs of test kit and processing are unknown. We estimated the costs of FIT test kit and processing based on the difference between gFOBT and FIT in a Dutch screening trial, and applied this difference to the cost of gFOBT in Ontario.

‡ We assumed complications with bleeding in 1.64 per 1,000 procedures, and complications with perforation in 0.85 per 1,000 procedures. In addition, we assumed 1/14,000 colonoscopies resulted in fatal complications.

§ CRC treatments were divided into three clinically relevant phases—initial, continuous and terminal care. The initial phase was defined as the first 12 months following diagnosis, the terminal phase was defined as the final 12 months of life, and the continuous phase was defined as all months between the initial and terminal phase. For patients surviving less than 24 months, the final 12 months were allocated to the terminal phase. The remaining months of observation were allocated to the initial phase.

### Cost-effectiveness analyses

For each screening strategy we estimated the number of QALYs gained and costs, compared to no screening. Strategies that were more costly and less effective than other strategies were ruled out by simple dominance. Strategies that were more costly and less effective than a mix of other strategies were ruled out by extended dominance. The remaining strategies that were not ruled out were referred to as “efficient” strategies. The incremental cost-effectiveness ratio (ICER) of an efficient strategy was determined by comparing its additional costs and health benefits to those of the next less costly and less effective efficient strategy.

### Sensitivity analyses

We performed several sensitivity analyses assuming: 1) dependency of test results between screening rounds (74% of large adenomas could not be detected because they did not bleed [33]); 2) half and double the base case rate of colonoscopy complications; 3) 25% increased CRC relative survival; 4) FIT unit costs of 43.87 CAN$ (based on the difference in reimbursement rate between FIT and gFOBT in the US Medicare program[34]); 5) half and double the base case value for colonoscopy costs; 6) half and double the base case value for CRC treatment costs.

### Outcomes

The main outcomes of the analysis were QALYs and costs per 1,000 participants, and number of colonoscopies per 1,000 participants per year, compared to no screening. Costs and QALYs were discounted by 3% per year[35], the number of colonoscopies were undiscounted.

## Results

**Fig 1:**
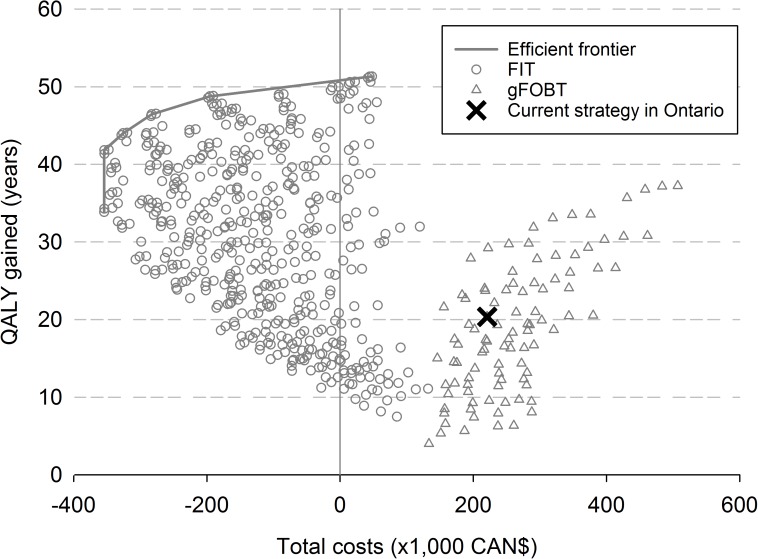
Discounted total costs and discounted QALYs gained, per 1,000 participants, of the gFOBT and FIT screening strategies compared to no screening. QALY: quality adjusted life year; gFOBT: guaiac fecal occult blood test; FIT: fecal immunochemical test. Current screening strategy in Ontario: biennial gFOBT, between age 50–74. Strategies are varied by age at starting screening, age at stopping screening, screening interval, and FIT cut-off level. The cost-effective strategies are connected by the efficient frontier. Costs (expressed in 2013 Canadian dollars) and QALYs are discounted by 3% per year.

The current screening strategy in Ontario, biennial gFOBT between age 50–74 years, yielded 20 QALYs at a cost of CAN$220,900 per 1,000 screening participants, compared to no screening (Fig 1). When colonoscopy capacity is not a limiting factor, increasing the screening age range to 40–85 years with annual gFOBT could provide a maximum of 37 QALYs at a cost of CAN$507,000 per 1,000 participants. For each gFOBT screening strategy there was a FIT strategy that provided more QALYs at lower costs, therefore FIT dominated gFOBT. The FIT strategies on the efficient frontier provided 34 to 51 QALYs, at a cost of -CAN$354,200 to -CAN$48,000 per 1,000 participants, compared to no screening. Assuming a willingness-to-pay threshold of CAN$50,000 per QALY gained, FIT every year between age 45–80 years would be the preferred strategy, providing 49 QALYs per 1,000 participants.

**Fig 2:**
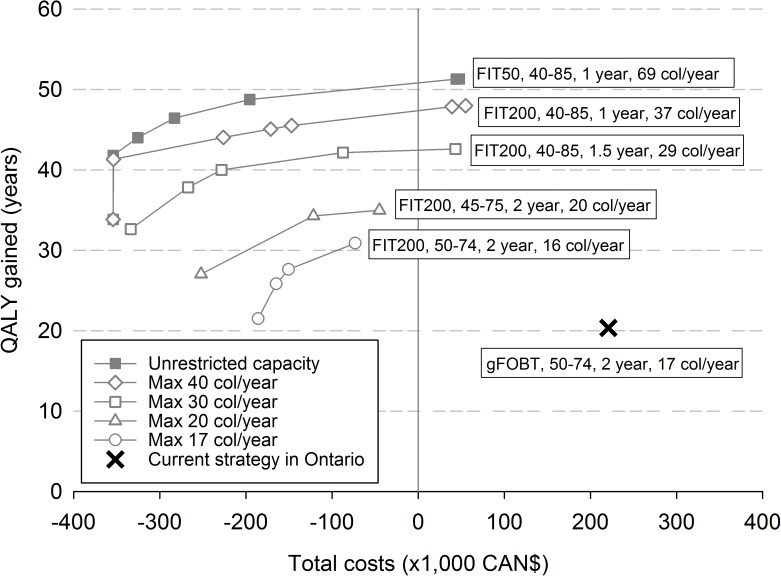
Efficient frontiers for different levels of colonoscopy capacity. Costs and QALYs gained per 1,000 participants, compared to no screening. QALY: quality adjusted life year; gFOBT: guaiac fecal occult blood test; FIT: fecal immunochemical test; Col/year: number of colonoscopies required per 1,000 participants per year. Strategies are varying by age at starting screening, age at stopping screening, screening interval, and FIT cut-off level. For each level of available colonoscopy capacity (maximal 17, 20, 30, 40 colonoscopies per 1,000 participants per year and unrestricted colonoscopy capacity) the cost-effective strategies are connected by their respective efficient frontier. The text boxes beside each frontier present the screening strategy (test, age range, interval and colonoscopy

With unrestricted colonoscopy capacity almost all cost-effective strategies used FIT with a cut-off level of 50 ng Hb/ml (Table 4, see Table 5 for intermediate outcomes). The number of colonoscopies required for the strategies on the efficient frontier ranged from 32 to 69 per 1,000 participants per year. This is a two- to four-fold increase over the colonoscopy demand of the current screening strategy in Ontario (17 colonoscopies per 1,000 participants per year). However, when colonoscopy capacity was restricted to 40, 30, 20, or 17 colonoscopies per year FIT remained more cost-effective than gFOBT. At 17 colonoscopies per 1,000 participants per year, biennial FIT with a cut-off level of 200 ng Hb/ml, between age 50–74 years still provided 31 QALYs at a cost of -CAN$73,200, compared to 20 QALYs at a cost of CAN$220,900 for gFOBT (Fig 2).

**Table 4:** Overview of the current gFOBT screening strategy in Ontario, and efficient FIT screening strategies, compared to no screening*. Outcomes per 1,000 participants.

Screen test	Start age (years)	Stop age (years)†	Interval (years)	Col/year (N)	QALYs (years)	Costs (CAN$)	ICER (CAN$)
Current screening strategy in Ontario							
gFOBT	50	75	2	16.9	20.3	220,915	dominated
Cost-effective screening strategies							
Unrestricted colonoscopy capacity							
FIT 50	55	75	1.5	31.6	33.8	-354,200	-10,500
FIT 50	50	80	1.5	40.9	41.8	-354,200	0
FIT 50	50	80	1	49.4	44.0	-325,600	13,000
FIT 50	45	80	1.5	48.8	46.5	-283,100	17,400
FIT 50	45	80	1	58.6	48.8	-195,600	37,800
FIT 50	40	80	1	68.7	51.3	44,300	95,100
FIT 50	40	85	1	69.1	51.3	48,000	132,300
Maximal 40 colonoscopies per 1,000 participants per year							
FIT 50	55	75	1.5	31.6	33.8	-354,200	-10,500
FIT 50	50	75	1.5	39.3	41.3	-353,900	5,000
FIT 75	45	75	1.5	39.2	44.0	-226,200	47,100
FIT 100	45	70	1	39.4	45.1	-171,300	52,600
FIT 150	45	80	1	36.2	45.5	-147,100	55,500
FIT 200	40	80	1	36.8	47.9	39,400	79,300
FIT 200	40	85	1	37.3	48.0	55,100	144,200
Maximal 30 colonoscopies per 1,000 participants per year							
FIT 50	55	70	1.5	29.1	32.6	-333,800	-10,200
FIT 75	50	70	1.5	29.9	37.8	-267,200	12,800
FIT 150	50	75	1	29.1	40.0	-228.300	17,900
FIT 200	45	70	1	28.4	42.2	-87,400	65,100
FIT 200	40	85	1.5	29.3	42.6	43,400	33,500
Maximal 20 colonoscopies per 1,000 participants per year							
FIT 50	60	75	3	19.9	27.0	-252,100	-9,300
FIT 200	50	75	1.5	19.6	34.3	-121,700	18,000
FIT 200	45	75	2	19.9	35.0	-45,100	107,900
Maximal 17 colonoscopies per 1,000 participants per year							
FIT 50	60	75	3	16.8	21.5	-185,800	-8,600
FIT 100	55	70	2	16.2	25.8	-164,800	4,800
FIT 150	55	70	1.5	16.5	27.6	-150,600	7,900
FIT 200	50	75	2	16.4	30.9	-73,200	23,700

Col/year: number of colonoscopies required per 1,000 participants per year; QALY: quality adjusted life year gained; ICER: incremental cost-effectiveness ratio. The number of colonoscopies per year are undiscounted. Costs and QALYs are discounted by 3% per year.

* Without screening, costs for management of CRC amount to $5.2 million and total QALY in the population in the cohort to 23 thousand QALY.

† Stop age of screening is not necessarily the age of last screening. The last age of screening depends on start age and interval and is the latest age that can be acquired with that start age and interval that still is below the stop age of screening. For example, screening every 1.5 years from age 55 results in a final screening to be performed at the age of 74.5 years.

**Table 5:** Undiscounted intermediate model outcomes per 1,000 participants, compared to no screening.

Screen test (age range, interval)	Total tests (N)	Positive tests (N)	Col/year (N)	CRC cases (N)	CRC deaths (N)	LYG (years)	QALYs (years)
Current screening strategy in Ontario							
gFOBT (50–74, 2)	10346	258	16.9	-12.6	-10.8	122.5	65.2
Cost-effective screening strategies (unrestricted colonoscopy capacity)							
FIT 50 (55–74.5, 1.5)	8989	491	31.6	-28.4	-17.9	194.0	109.3
FIT 50 (50–80, 1.5)	11695	609	40.9	-32.6	-20.2	225.8	130.3
FIT 50 (50–80, 1)	14563	725	49.4	-35.6	-20.9	235.4	136.8
FIT 50 (45–79.5, 1.5)	13094	659	48.8	-34.3	-20.7	240.6	141.5
FIT 50 (45–80, 1)	16107	779	58.6	-37.3	-21.6	250.9	148.3
FIT 50 (40–80, 1)	17441	822	68.7	-38.6	-22.0	260.5	154.8
FIT 50 (40–85, 1)	17791	839	69.1	-38.9	-22.2	261.4	154.9

Col/year: number of colonoscopies required per 1,000 participants per year; CRC: colorectal cancer; LYG: life year gained; QALY: quality adjusted life year gained.

### Sensitivity analyses

The more favorable cost-effectiveness of FIT compared to gFOBT screening strategies was robust to alterations in our model assumptions. None of the sensitivity analyses resulted in a gFOBT strategy on the efficient frontier (S2 Table). Varying colonoscopy and treatment costs had the largest impact on cost-effectiveness.

### Interpretation

Our study shows that compared to the current CRC screening strategy in Ontario (biennial gFOBT between age 50–74 years), replacing gFOBT by FIT with a cut-off level of 200 ng Hb/ml provides more QALYs at lower costs, without increasing the number of colonoscopies required. If the colonoscopy capacity were expanded greater health benefits and cost-reductions could be achieved by lowering the FIT cut-off level and shortening the screening interval from biennial to annual. Without restriction in colonoscopy capacity and assuming a willingness-to-pay threshold of CAN$50,000 per QALY, FIT at a cut-off of 50 ng/ml between age 40–80 years with a 1 year interval was the most effective strategy providing 47 QALYs compared to no screening.

The fact that screening FIT is less costly than gFOBT (and even cost-saving compared to no screening) results from the combination of increased sensitivity for adenomas and high costs for CRC treatment. GFOBT mainly detects CRC. While early detection of CRC is associated with a reduction in CRC mortality, the costs of CRC treatment are not substantially reduced. On the other hand FIT, even at the cut-off level of 200 ng Hb/ml, is more than twice as sensitive for large adenomas than gFOBT,[24] and is associated with prevention of more CRC and associated treatments. At the 200 cut-off level, the specificity of FIT is similar to gFOBT resulting in similar colonoscopy demand.[24]

Most previous cost-effectiveness analyses found FIT screening to be cost-effective, but FIT was generally also more costly than gFOBT.[32, 36–41] However, most studies used what are now outdated estimates of CRC treatment costs[42] and considered a single, or a limited number of screening strategies. Our findings are in line with the study by Heitman et al. which reported FIT screening to be more effective and less costly than gFOBT.[43] Heitman et al. used an indirect method to estimate current CRC treatment costs in Canada. In our analysis we used recent CRC treatment data as observed with a fully allocated costing approach and included costs of recently introduced biologic therapies (manuscript in preparation).

Our study adds to the study of Heitman et al in several ways. First, multiple models corroborating the same conclusion strengthen the confidence in that conclusion, especially when the models differ in their structure for the natural history of CRC (e.g. MISCAN assumes sensitivity of FIT to depend on time to clinical diagnosis and assumes improved prognosis of screen-detected cancers vs clinically diagnosed cancers). Second, we explored a much wider range of gFOBT and FIT screening strategies than Heitman (different start and stop age, screening intervals and FIT cut-off level). This analysis shows that FIT is always the preferred strategy across this whole range of strategies, but more importantly this approach allows selection of the optimal strategy for Ontario. In addition, we considered different levels of available colonoscopy capacity to see if FIT would still be the preferred strategy if colonoscopy capacity is limited. Our results clearly indicate that even at lower colonoscopy capacity levels, it is still most efficient to use FIT-based screening, albeit at higher cut-offs.

Our study should be interpreted in light of its strengths and limitations. First, there is considerable uncertainty in assumptions used in the model. Several assumptions could not be directly estimated using Canadian information and were therefore based on international data. We evaluated the impact of uncertainty on several parameters in one-way sensitivity analyses and found that our results were robust to these assumptions. One of the most uncertain assumptions is that all CRCs arise from adenoma precursors. We considered a sensitivity analysis with the assumption that 74% of large adenomas did not bleed (and were therefore undetectable) by gFOBT and FIT[33], which did not greatly affect the relative cost-effectiveness of FIT compared to gFOBT. We did not perform a probabilistic sensitivity analysis. Given the large number of strategies that would need to be evaluated, such an analysis would require a huge computational effort. We prioritized the large number of strategies over the probabilistic sensitivity analysis, because we were primarily interested in the comparison between different gFOBT and FIT screening strategies allowing for varying screening age ranges, intervals and FIT cut-off levels. Given the similar nature of gFOBT and FIT screening, many uncertainties in model parameters influence both gFOBT and FIT in a similar way and will therefore not influence the comparative effectiveness of FIT versus gFOBT. The difference in performance between both tests is mainly driven by the differences in test characteristics for which there is very convincing evidence that FIT outperforms gFOBT screening from several studies.[24, 26] This is also the reason that it is the preferred method of screening according to the European guidelines.[44] We therefore don’t expect that the conclusions of this study would change if we had performed a probabilistic sensitivity analysis.

Second, we assumed perfect adherence to screening, follow-up and surveillance, in order to represent the cost-effectiveness for participants who follow program recommendations. On a population level, screening adherence will be less than 100%, which will impact the cost-effectiveness ratios. However, it has been demonstrated that adherence to FIT is greater than to gFOBT screening.[25, 26] Therefore the difference in cost-effectiveness between the two tests is likely to be even greater when screening adherence is taken into account.

Finally, we did not explicitly model distinct pathways for traditional and sessile serrated adenomas. The average time it takes for an adenoma to develop into CRC was calibrated to the UK flexible sigmoidoscopy screening trial which included both traditional and sessile serrated adenomas. Both adenoma types are therefore included in the modelled mix of slow and rapid progressing lesions. Our conclusion would only be influenced by not explicitly modeling the serrated polyp pathway if the sensitivity for serrated adenomas would differ between FIT and gFOBT *and* these lesions would have higher malignant potential than adenomas in general. Limited evidence suggests that FIT might be less sensitive for serrated polyps than for adenomas, because they are often flat and therefore less likely to bleed. However, given the similar nature of gFOBT, this test is expected to be affected similarly.

This study has been performed in the setting of the ColonCancerCheck program in Ontario, Canada. In addition to Ontario, there are a number of regions around the world that use gFOBT in their CRC screening programs.[5, 6] Provided that the relative difference between the costs of screening tests and CRC treatment is not radically different from Ontario, the results from this study can be generalized to these other jurisdictions.

In conclusion, we found FIT to be more effective and less costly than gFOBT screening in average risk individuals for a wide range of screening strategies. This conclusion was robust to a wide range of assumptions. The optimal FIT strategy depends on the available colonoscopy capacity. Compared to gFOBT screening, introducing FIT at a high cut-off level could increase the health benefits of a CRC screening program without considerably increasing colonoscopy demand.

## Supporting information

S1 Appendix MISCAN-Colon model description. (DOCX)

S1 Table Simulated (Observed) Positivity Rates and Detection Rates per 100 Screened Individuals (Highest Grade Finding per Individual) for FIT at Cutoff Levels of 50, 75, 100, 150, and 200 ng/mL in the First Screening Round of the Dutch Trials. ^a^ Advanced adenoma was defined as an adenoma ≥10 mm or with histology showing either a ≥25% villous component or high-grade dysplasia in the trials. In the model, adenomas are classified by size only and advanced adenomas were defined as ≥10 mm.
(DOCX)

S2 Table Outcomes from the base case and sensitivity analyses (per 1,000 participants). Col/year: colonoscopies per year; QALY: quality adjusted life years; ICER: incremental cost-effectiveness ratio.
The number of colonoscopies per year are undiscounted.
Costs (expressed in 2013 Canadian dollars) and QALYs are discounted by 3% per year.
(DOCX)

## Abbreviations

CRC: colorectal cancer
gFOBT: guaiac fecal occult blood test
FIT: fecal immunochemical test
QALY: quality adjusted life year gained
ICER: incremental cost-effectiveness ratio
