# Supplementary material for: Harms, benefits and costs of fecal immunochemical testing versus guaiac fecal occult blood testing for colorectal cancer screening
Source: PLoS One. 2017 Mar 15;12(3):e0172864. doi: 10.1371/journal.pone.0172864 (PMC5351837; doi:10.1371/journal.pone.0172864)
Supplement: S1 Appendix — (DOCX) [file pone.0172864.s001.docx]

## S1 Appendix: MISCAN-Colon model description

**OUTLINE**

# Model Overview Page 1

# Demography part Page 2

*Natural history part Page 2*

*Screening part Page 3*

*Integration of the three components Page 3*

Model Quantification Page 6

*Demography parameters Page 6*

*Natural history parameters Page 6*

*Screen parameters Page 9*

*Ontario specific calibration data Page 11*

**MODEL OVERVIEW**

The MISCAN-Colon model is a semi-Markov microsimulation model, that has been programmed in Borland Delphi 7 Enterprise. The population is simulated individual by individual, and each person can evolve through discrete disease states. However, instead of modeling yearly transitions with associated transition probabilities, the MISCAN-Colon model generates durations in states. This improves model performance and with the assumption of exponential distributions of durations in each state this way of simulating leads to similar results as a Markov model with yearly transition probabilities. The advantage of the MISCAN approach is that durations in a certain state do not necessarily need to be a discrete value, but can be continuous. MISCAN uses the Monte Carlo method to simulate all events in the program. Possible events are birth and death of a person, adenoma incidence and transitions from one state of disease to another.

The basic structure of MISCAN-Colon is illustrated in Figure A1.1. This figure demonstrates that MISCAN-Colon consists of three parts:

- demography part
- natural history part
- screening part

These parts are not physically separated in the program, but it is useful to consider them separately.

**Figure A1.1:** Structure of MISCAN-Colon

*DEMOGRAPHY PART*

The demography part of the model simulates individual life histories without colorectal cancer to form a population. For each person, a date of birth and a date of death from other causes than colorectal cancer are simulated. The distribution of births and deaths can be adjusted to represent the population simulated. For example, a population of Caucasian females will have higher death ages than a population of African American males.

*NATURAL HISTORY PART*

The Natural History part of MISCAN-Colon simulates the development of colorectal cancer in the population. We assume all colorectal cancers develop according to the adenoma-carcinoma sequence of Morson[1] and Vogelstein[2] (Figure A1.2). For each individual in the simulated population a personal risk index is generated. Subsequently, adenomas are generated in the population according to each personal risk index and an age specific incidence rate of adenomas. This results in no adenomas for most persons and one or more adenomas for others. The distribution of adenomas over the colon and rectum is simulated according to the observed distribution of colorectal cancer incidence. Each of the adenomas can independently develop into colorectal cancer. Adenomas can progress in size from small (≤5 mm) to medium (6-9 mm) to large (10≥ mm). Most adenomas will never develop into cancer (non-progressive adenomas), but some (progressive adenomas) may eventually become malignant, transforming to a stage I cancer. The cancer may then progress from stage I to stage IV. In every stage there is a chance the cancer will be diagnosed because of symptoms. The survival after clinical diagnosis depends on the stage of the cancer.

**Figure A1.2:** Adenoma and cancer stages in the MISCAN-Colon model. Cancer stages correspond to the American Joint Committee on Cancer / International Union Against Cancer staging system for colorectal cancer. Adenomas are categorized by size. The size-specific prevalence of adenomas as well as the proportion of adenomas that ever develop into cancer is dependent on age (estimates of model parameters are presented in the section “MODEL QUANTIFICATION” on page 6).

*SCREENING PART*

Screening interrupts the development of CRC. With screening, adenomas may be detected and removed and cancers may be found, usually in an earlier stage than with clinical diagnosis. In this way screening can prevent CRC incidence or CRC death. The life-years gained by screening are calculated by comparing the model-predicted life-years lived in the population with and without screening. The effects of different screening policies can be compared by applying them to identical natural histories.

*INTEGRATION OF THE THREE MODEL COMPONENTS*

For each individual, the demography part of the model simulates a time of birth and time of death from other causes than colorectal cancer, creating a life history without colorectal cancer (top line in Figure A1.3a). Subsequently adenomas are simulated for that individual. For most individuals no adenomas are generated and for others one or more. In the example in Figure A1.3, the person gets two adenomas (2nd and 3rd line in Figure A1.3a). The first adenoma arises at a certain age, grows into 6-9 mm and eventually becomes larger than 10 mm. However, this adenoma does not become cancer before the death of the person. The second adenoma is a progressive adenoma. After having grown to 6-9 mm, the adenoma transforms into a malignant carcinoma, causing symptoms and diagnosis and eventually resulting in an earlier death from CRC. The life history without CRC and the development of the two adenomas in Figure A1.3 together lead to the combined life history with CRC depicted in the bottom line. Because this person dies from colorectal cancer before he dies from other causes, his death age is adjusted accordingly.

**Figure A1.3a:** Modeling natural history into life history

After the life history of a person is adjusted for colorectal cancer, the history will now be adjusted for the effects of screening. The effect of screening on life history is explained in Figure A1.3b.The top line in this figure is the combined life history for colorectal cancer from Figure A1.3a. The development of the separate adenomas is repeated in the second and third line. In this picture there is one screening intervention. During the screening both prevalent adenomas are detected and removed. This results in a combined life history for colorectal cancer and screening (bottom line). From the moment of screen detection the adenomas are removed and this individual becomes adenoma and carcinoma free. He does not develop cancer because the precursor lesion has been removed. Therefore the person dies at the moment of death from other causes and the effect of screening is the difference in life-years in the situation with and without screening. Of course many other possibilities could have occurred: a person could have developed new adenomas after the screening moment, or an adenoma could have been missed by the screening test, but in this example this individual really benefited from the screening intervention.

**Figure A1.3b:** Modeling screening into life history

**MODEL QUANTIFICATION**

*DEMOGRAPHY PARAMETERS*

For this analysis we simulated a cohort of individuals born in 1974, which resulted in individuals aged 40 in 2014 (the youngest age to start screening in the simulated screening strategies). The all-cause mortality estimates used in the model were derived from the 2009-2011 Ontario life tables.[3]

*NATURAL HISTORY PARAMETERS*

The parameters for the natural history model that could not be directly estimated from data or fit to reference data, were established based on expert opinion. At two expert meetings at the NCI on June 5–7, 1996, and May 12–13, 1997, a model structure was devised in agreement with the currently accepted model of the adenoma–carcinoma sequence. It was assumed that all cancers are preceded by adenomas.

The average duration between onset of a progressive adenoma and the transition to preclinical cancer was calibrated to data from the UK flexible sigmoidoscopy screening trial.[4] The duration of cancer in preclinical stages was estimated based on the results of three large randomized controlled screening trials.[5] This resulted in an average duration of 2.5 years, 2.5 year, 3.7 years, and 1.5 year, for stages I-IV respectively. The total duration from initial development of an adenoma to clinical diagnosis of CRC in cancer cases is on average 17.2 years; 12.5 years from adenoma incidence to preclinical CRC, and 4.7 years from preclinical to clinical CRC. The total duration of preclinical cancer until clinical diagnosis (4.7 years) is shorter than the sum of the average durations of the individual cancer stages. This is because some cancers will be clinically diagnosed before reaching stage IV, and because some individuals will die of other causes before clinical diagnosis. All durations were governed by an exponential probability distribution. Durations in each of the invasive cancer stages as well as durations in the stages of the noninvasive adenomas were assumed to be 100% associated with each other, but the durations in invasive stages as a whole were independent of durations in noninvasive adenoma stages that precede cancer. These assumptions resulted in an exponential distribution of the total duration of progressive noninvasive adenomas and of the total duration of preclinical cancer, which has also been used in other cancer screening models.[6, 7]

It was assumed that 30% of the cancers arise from adenomas of 6–9 mm and that 70% arise from larger adenomas. Initially, the preclinical incidence of progressive adenomas was chosen to reproduce the colorectal cancer incidence by age, stage, and localization in Ontario in 2001, which was before the introduction of CRC screening.[8] The size distribution of adenomas over all ages was assumed to be 56% for stages less than or equal to 5 mm, 24% for stages 6–9 mm, and 20% for stages greater than or equal to 10 mm.[9-18] The preclinical incidence of non-progressive adenomas that will never grow into cancer was varied until the simulated prevalence of all adenomas was in agreement with data from autopsy studies.[9-18] The anatomic site distribution of both progressive and non-progressive adenomas and thus of preclinical and clinical cancers is assumed to be equal to the site distribution of colorectal cancers in Ontario in 2001.[8] Because stage-specific data on colorectal cancer relative survival were not available for Ontario, we assumed the same age- and stage specific CRC relative survival as observed in the Surveillance, Epidemiology, and End-Results database in the US, in the period 2000-2003.[19] Table A1.1 contains a summary of the model input values and its data-sources.

**Table A1.1:** Main natural history assumptions in the MISCAN-Colon model

| **Model parameter** | **Value** | | | **Source** |
| --- | --- | --- | --- | --- |
| Distribution of risk for adenomas over the general population | Gamma distributed, mean 1, variance gender dependent (2.21 for males, 3.15 for females) | | | Fit to multiplicity distribution of adenomas in autopsy studies [9-18] and to cancer incidence in Ontario in 2001.[8] |
| Adenoma incidence in general population | *Age*  *(years)* | *Males average risk* | *Females average risk* | Fit to adenoma prevalence in autopsy studies,[9-18] and to cancer incidence in Ontario in 2001.[8] |
|  | 0-19 | 0.2% | 0.2% |  |
|  | 20-24 | 0.1% | 0.3% |  |
|  | 25-29 | 0.1% | 0.3% |  |
|  | 30-34 | 0.3% | 0.4% |  |
|  | 35-39 | 1.4% | 0.5% |  |
|  | 40-44 | 2.4% | 3.6% |  |
|  | 45-49 | 1.9% | 2.1% |  |
|  | 50-54 | 1.6% | 1.9% |  |
|  | 55-59 | 5.1% | 1.5% |  |
|  | 60-64 | 4.5% | 1.7% |  |
|  | 65-69 | 4.6% | 1.9% |  |
|  | 70-74 | 5.4% | 2.5% |  |
|  | 75-79 | 5.5% | 7.6% |  |
|  | 80-84 | 5.2% | 7.9% |  |
|  | 85-100 | 4.7% | 8.1% |  |
| Probability that a new adenoma is progressive | Dependent on gender and age at onset.  *Males:*  0–45 years: linearly increasing from 0 to 37%  45–65 years: linearly increasing from 37% to 76%  65–100 years: linearly increasing from 76% to 99%  *Females:*  0–45 years: linearly increasing from 0 to 23%  45–65 years: linearly increasing from 23% to 98%  65–100 years: linearly decreasing from 98% to 64% | | | Fit to adenoma prevalence in autopsy studies,[9-18] and to cancer incidence in Ontario in 2001.[8] |
| Regression of adenomas | No significant regression of adenomas | | | Expert opinion |
| Average duration of preclinical cancer by stage | Stage I: 2.5 years  Stage II: 2.5 year  Stage III: 3.7 years  Stage IV: 1.5 year | | | Estimated from FOBT trials.[5] |
| Average and interquartile range (IQR) of durations in the adenoma stage and cancer stage among clinically diagnosed cancer cases | - Adenoma incidence to preclinical cancer: 12.5 (4-18) years.  - Preclinical cancer to cancer diagnosis: 4.7 (1-7) years.  - Adenoma incidence to cancer diagnosis: 17.2 (9-24) years.  The total duration of preclinical cancer until clinical diagnosis (4.7 years) is shorter than the sum of the average durations of the individual cancer stages. This is because some cancers will be clinically diagnosed before reaching stage IV, and because some individuals will die of other causes before clinical diagnosis. | | | Adenoma duration estimated from a once only sigmoidoscopy study[4], cancer duration estimated from FOBT trials.[5] |
| Percent of non-progressive adenomas that stay 6-9mm | 25% | | | Fit to size distribution of adenomas in autopsy studies: [9-18]  1-5mm: 56%  6-9 mm: 24%  10+ mm: 20% |
| Percent of non-progressive adenoma that become 10mm or larger | 75% | | | Fit to size distribution of adenomas in autopsy studies: [9-18]  1-5mm: 56%  6-9 mm: 24%  10+ mm: 20% |
| Percent of cancers that develops from 6-9mm adenoma and from 10+mm adenoma | 30% of cancer develops from 6-9 mm, 70% from 10+mm | | | Expert opinion |
| Localization distribution of adenomas and cancer | Males: rectum: 24%, colon: 76%  Females: rectum: 16%, colon: 84% | | | Estimated from Ontario cancer incidence in 2001.[8] |
| 10-year survival after clinical diagnosis of CRC | Dependent on age and stage at diagnosis, and localization | | | Estimated from the Surveillance, Epidemiology, and End-Results database in the US, in the period 2000-2003.[19] |

*SCREEN PARAMETERS*

The test characteristics of the gFOBT (Hemoccult II) were based on a prior calibration of the MISCAN model to three large gFOBT screening trials,[5] and the test characteristics of the fecal immunochemical test (FIT, OC-Sensor Micro; Eiken Chemical Co, Tokyo, Japan) were based on two screening trials from the Netherlands (Table A1.2).[20] It was assumed that the probability a CRC bleeds, and thus the sensitivity of stool tests for CRC, depends on the time until clinical diagnosis, hence the distinction between ‘early’ and ‘late’ preclinical CRC. This is to be expected when cancers that bleed do so increasingly over time, starting with occult blood loss and progressing to clinically visible bleeding.[5]

The test characteristics of colonoscopy were based on a systematic review of tandem colonoscopy studies.[21] It was assumed that 95% of all colonoscopies reached the proximal end of the cecum; the reach of the remaining 5% was distributed linearly over the colon and rectum.[22] Hyperplastic polyps, which do not follow the adenoma-carcinoma sequence, are not modeled explicitly but are reflected in the specificity of colonoscopy.[23] Additional biopsy costs were assumed for procedures where biopsies were performed and in which, in retrospect, no adenomas were detected. We modeled a fatal complication rate for colonoscopies of 1/14,000 procedures.[24] The sensitivity and specificity of all screening tests were assumed to be independent of screening round and age.

The stage-specific survival of patients with screen-detected cancer was based on a previous analysis,[5] and was more favorable than the survival after diagnosis in the same stage without screen-detecting. Removal of an adenoma always prevents development of any subsequent cancer that may have arisen from this adenoma.

### Table A1.2: Screen test characteristics

| **Screen test** | **Sensitivity^*^ (%)** | | | | | **Specificity**  **(%)** |
| --- | --- | --- | --- | --- | --- | --- |
|  | **Adenoma ≤5mm** | **Adenoma**  **6-9mm** | **Adenoma ≥10mm** | **CRC early Preclinical^†^** | **CRC late Preclinical^†^** |  |
| gFOBT | 2 | 3 | 8 | 20 | 52 | 98 |
| FIT50 | 4 | 15 | 37 | 52 | 83 | 96 |
| FIT75 | 3 | 9 | 31 | 48 | 81 | 97 |
| FIT100 | 2 | 7 | 28 | 43 | 77 | 98 |
| FIT150 | 2 | 5 | 25 | 41 | 76 | 98 |
| FIT200 | 1 | 4 | 21 | 40 | 76 | 99 |
| Colonoscopy | 75 | 85 | 95 | 95 | 95 | 90‡ |

CRC: colorectal cancer; gFOBT: guaiac fecal occult blood test (Hemoccult II); FIT: fecal immunochemical test, at a cut-off level of 50, 75, 100, 150 and 200 ng Hb/ml.

* It is assumed that small adenomas do not bleed and cannot be detected by gFOBT and FIT. The sensitivity of FOBT and FIT for adenomas ≤5 mm is based on the false-positive rate (i.e., 1 - specificity).

† It was assumed that the probability a CRC bleeds and thus the sensitivity of gFOBT and FIT for CRC depends on the time until clinical diagnosis.[5]

‡ Hyperplastic polyps, which do not follow the adenoma-carcinoma sequence, are not modeled explicitly but are reflected in the specificity of colonoscopy.[23] Additional biopsy costs were assumed for procedures where biopsies were performed and in which, in retrospect, no adenomas were detected.

### *ONTARIO SPECIFIC CALIBRATION DATA*

### The MISCAN model was made Ontario specific by calibrating the CRC incidence rate and stage distribution to observed data from Ontario (Table A1.3 and Table 1.4). The data was from the year 2001, which was before the widespread introduction of CRC screening. Because sex specific data for the stage distribution was not available, we assumed equal stage distribution for both sexes. In addition, the stage distribution was only available for stages “localized”, “regional”, and “distant”. Because the model simulates four CRC stages, instead of three, we redistributed the cases in the localized and regional stages over stage I-III in the model, based on the observed CRC stage distribution in the US.

### Table A1.3: Number of CRC cases observed in Ontario in 2001.

| **Age group** | **Males** | | | **Females** | | |
| --- | --- | --- | --- | --- | --- | --- |
|  | **Colon + rectosigmoid (N)** | **Rectum (N)** | **Population size (N)** | **Colon + rectosigmoid (N)** | **Rectum (N)** | **Population size (N)** |
| <20 | 2 | 0 | 1590805 | 1 | 0 | 1522107 |
| 20-24 | 3 | 0 | 400613 | 0 | 1 | 383121 |
| 25-29 | 7 | 0 | 407204 | 4 | 0 | 399024 |
| 30-34 | 8 | 1 | 448576 | 8 | 2 | 447194 |
| 35-39 | 30 | 9 | 526034 | 24 | 6 | 518026 |
| 40-44 | 47 | 22 | 501957 | 53 | 12 | 503011 |
| 45-49 | 96 | 33 | 434736 | 92 | 29 | 446427 |
| 50-54 | 160 | 75 | 392233 | 126 | 37 | 399073 |
| 55-59 | 229 | 104 | 297672 | 198 | 47 | 302557 |
| 60-64 | 330 | 124 | 236736 | 250 | 51 | 249420 |
| 65-69 | 440 | 145 | 209194 | 324 | 52 | 226257 |
| 70-74 | 500 | 137 | 181628 | 378 | 83 | 210691 |
| 75-79 | 467 | 120 | 132687 | 438 | 70 | 183922 |
| 80-84 | 279 | 69 | 73398 | 373 | 66 | 120009 |
| 85+ | 195 | 43 | 33615 | 363 | 48 | 70034 |

### Table A1.4: Stage distribution^*^ of CRC diagnosed in Ontario in 2001.

| **Age group** | **Colon + rectosigmoid** | | | **Rectum** | | |
| --- | --- | --- | --- | --- | --- | --- |
|  | **Localized (%)** | **Regional**  **(%)** | **Distant (%)** | **Localized (%)** | **Regional**  **(%)** | **Distant (%)** |
| 50-54 | 33.8 | 36.1 | 27.1 | 25.5 | 41.2 | 26.5 |
| 55-59 | 32.4 | 40.3 | 23.7 | 20.1 | 41.5 | 35.6 |
| 60-64 | 34.3 | 36.9 | 26.3 | 24.0 | 45.3 | 26.0 |
| 65-69 | 31.7 | 38.2 | 26.0 | 23.3 | 37.1 | 32.7 |
| 70-74 | 40.1 | 31.3 | 23.5 | 26.9 | 45.2 | 23.7 |

### ^*^ Because data were not available for males and females separately, we assumed equal stage distribution in both sexes.

### REFERENCES

1. Morson B. President's address. The polyp-cancer sequence in the large bowel. *Proc R Soc Med*. 1974;67(6 Pt 1):451-7.

2. Vogelstein B, Fearon ER, Hamilton SR, Kern SE, Preisinger AC, Leppert M, et al. Genetic alterations during colorectal-tumor development. *N Engl J Med*. 1988;319(9):525-32.

3. Statistics Canada. Life Tables, Canada, Provinces and Territories 2009 to 2011. Available at: <http://www.statcan.gc.ca/pub/84-537-x/84-537-x2013005-eng.htm>. Accessed: December 22, 2014.

4. Atkin WS, Edwards R, Kralj-Hans I, Wooldrage K, Hart AR, Northover JM, et al. Once-only flexible sigmoidoscopy screening in prevention of colorectal cancer: a multicentre randomised controlled trial. *Lancet*. 2010;375(9726):1624-33.

5. Lansdorp-Vogelaar I, van Ballegooijen M, Boer R, Zauber A, Habbema JD. A novel hypothesis on the sensitivity of the fecal occult blood test: Results of a joint analysis of 3 randomized controlled trials. *Cancer*. 2009;115(11):2410-9.

6. Gyrd-Hansen D, Sogaard J, Kronborg O. Analysis of screening data: colorectal cancer. *Int J Epidemiol*. 1997;26(6):1172-81.

7. Launoy G, Smith TC, Duffy SW, Bouvier V. Colorectal cancer mass-screening: estimation of faecal occult blood test sensitivity, taking into account cancer mean sojourn time. *Int J Cancer*. 1997;73(2):220-4.

8. Statistics Canada. Table 103-0550 - New cases for ICD-O-3 primary sites of cancer (based on the July 2011 CCR tabulation file), by age group and sex, Canada, provinces and territories. Available at: <http://www5.statcan.gc.ca/cansim/a01?lang=eng>. Accessed: December 22, 2014.

9. Arminski TC, McLean DW. Incidence and Distribution of Adenomatous Polyps of the Colon and Rectum Based on 1,000 Autopsy Examinations. *Dis Colon Rectum*. 1964;7:249-61.

10. Blatt L. Polyps of the Colon and Rectum: Incidence and Distribution. *Dis Colon Rectum*. 1961;4:277-82.

11. Bombi JA. Polyps of the colon in Barcelona, Spain. An autopsy study. *Cancer*. 1988;61(7):1472-6.

12. Chapman I. Adenomatous polypi of large intestine: incidence and distribution. *Ann Surg*. 1963;157:223-6.

13. Clark JC, Collan Y, Eide TJ, Esteve J, Ewen S, Gibbs NM, et al. Prevalence of polyps in an autopsy series from areas with varying incidence of large-bowel cancer. *Int J Cancer*. 1985;36(2):179-86.

14. Jass JR, Young PJ, Robinson EM. Predictors of presence, multiplicity, size and dysplasia of colorectal adenomas. A necropsy study in New Zealand. *Gut*. 1992;33(11):1508-14.

15. Johannsen LG, Momsen O, Jacobsen NO. Polyps of the large intestine in Aarhus, Denmark. An autopsy study. *Scand J Gastroenterol*. 1989;24(7):799-806.

16. Rickert RR, Auerbach O, Garfinkel L, Hammond EC, Frasca JM. Adenomatous lesions of the large bowel: an autopsy survey. *Cancer*. 1979;43(5):1847-57.

17. Vatn MH, Stalsberg H. The prevalence of polyps of the large intestine in Oslo: an autopsy study. *Cancer*. 1982;49(4):819-25.

18. Williams AR, Balasooriya BA, Day DW. Polyps and cancer of the large bowel: a necropsy study in Liverpool. *Gut*. 1982;23(10):835-42.

19. Surveillance Epidemiology and End Results (SEER) Program. SEER*Stat Database: Incidence - SEER 9 Regs Public Use. Nov 2003 Sub (1973-2001), DCCPS, Surveillance Research Program, Cancer Statistics Branch. Based on the November 2003 submission. Bethesda, MD: National Cancer Institute. Available at: <http://www.seer.cancer.gov>. Accessed: December 22, 2014.

20. Goede SL, van Roon AH, Reijerink JC, van Vuuren AJ, Lansdorp-Vogelaar I, Habbema JD, et al. Cost-effectiveness of one versus two sample faecal immunochemical testing for colorectal cancer screening. *Gut*. 2013;62(5):727-34.

21. van Rijn JC, Reitsma JB, Stoker J, Bossuyt PM, van Deventer SJ, Dekker E. Polyp miss rate determined by tandem colonoscopy: a systematic review. *Am J Gastroenterol*. 2006;101(2):343-50.

22. Cotterill M, Gasparelli R, Kirby E. Colorectal cancer detection in a rural community. Development of a colonoscopy screening program. *Can Fam Physician*. 2005;51:1224-8.

23. Morson BC. Precancerous lesions of the colon and rectum. Classification and controversial issues. *Jama*. 1962;179:316-21.

24. Rabeneck L, Paszat LF, Hilsden RJ, Saskin R, Leddin D, Grunfeld E, et al. Bleeding and perforation after outpatient colonoscopy and their risk factors in usual clinical practice. *Gastroenterology*. 2008;135(6):1899-906, 906 e1.

25. Zorzi M, Barca A, Falcini F, Grazzini G, Pizzuti R, Ravaioli A, et al. Screening for colorectal cancer in Italy: 2005 survey. *Epidemiol Prev*. 2007;31(2-3 Suppl 2):49-60.

26. Zauber AG, Lansdorp-Vogelaar I, Wilschut J, et al. Cost-effectiveness of DNA Stool Testing to Screen for Colorectal Cancer. Rockville, MD: Agency for Healthcare Research and Quality, 2007. Available at: <https://www.cms.gov/Medicare/Coverage/DeterminationProcess/downloads/id52TA.pdf>. Accessed: December 22, 2014.
