# Supplementary material for: Harms, benefits and costs of fecal immunochemical testing versus guaiac fecal occult blood testing for colorectal cancer screening
Source: PLoS One. 2017 Mar 15;12(3):e0172864. doi: 10.1371/journal.pone.0172864 (PMC5351837; doi:10.1371/journal.pone.0172864)
Supplement: S1 Table — a Advanced adenoma was defined as an adenoma ≥10 mm or with histology showing either a ≥25% villous component or high-grade dysplasia in the trials. In the model, adenomas are classified by size only and advanced adenomas were defined as ≥10 mm. (DOCX) [file pone.0172864.s002.docx]

**S1 Table: Simulated (Observed) Positivity Rates and Detection Rates per 100 Screened Individuals (Highest Grade Finding per Individual) for FIT at Cutoff Levels of 50, 75, 100, 150, and 200 ng/mL in the First Screening Round of the Dutch Trials**

| Test | Positivity rate | No neoplasia despite FIT result above cutoff level | Nonadvanced adenomas | Advanced adenomas[^a^](http://www.gastrojournal.org/article/S0016-5085(11)00984-X/fulltext#title-footnote-tblfn3) | CRC |
| --- | --- | --- | --- | --- | --- |
| FIT 200 | 3.7 (3.7) | 1.3 (1.3) | 0.48 (0.48) | 1.54 (1.54) | 0.39 (0.39) |
| FIT 150 | 4.4 (4.4) | 1.6 (1.6) | 0.59 (0.58) | 1.78 (1.82) | 0.40 (0.40) |
| FIT 100 | 5.3 (5.3) | 2.1 (2.1) | 0.83 (0.80) | 1.98 (2.01) | 0.42 (0.42) |
| FIT 75 | 6.4 (6.4) | 2.7 (2.7) | 0.99 (1.02) | 2.30 (2.27) | 0.45 (0.45) |
| FIT 50 | 8.4 (8.4) | 3.6 (3.7) | 1.57 (1.54) | 2.73 (2.71) | 0.48 (0.48) |

[^a^](http://www.gastrojournal.org/article/S0016-5085(11)00984-X/fulltext#back-tblfn3) Advanced adenoma was defined as an adenoma ≥10 mm or with histology showing either a ≥25% villous component or high-grade dysplasia in the trials. In the model, adenomas are classified by size only and advanced adenomas were defined as ≥10 mm.
