# Supplementary material for: Harms, benefits and costs of fecal immunochemical testing versus guaiac fecal occult blood testing for colorectal cancer screening
Source: PLoS One. 2017 Mar 15;12(3):e0172864. doi: 10.1371/journal.pone.0172864 (PMC5351837; doi:10.1371/journal.pone.0172864)
Supplement: S2 Table — Col/year: colonoscopies per year; QALY: quality adjusted life years; ICER: incremental cost-effectiveness ratio. The number of colonoscopies per year are undiscounted. Costs (expressed in 2013 Canadian dollars) and QALYs are discounted by 3% per year. (DOCX) [file pone.0172864.s003.docx]

**S2 Table: Outcomes from the base case and sensitivity analyses (per 1,000 participants).**

**Base case**

| **Screen test** | **Start age** | **Stop age** | **Interval** | **Col/year** | **QALY (years)** | **Costs ($)** | **ICER ($)** |
| --- | --- | --- | --- | --- | --- | --- | --- |
| Current screening strategy in Ontario | | | | |  |  |  |
| gFOBT | 50 | 74 | 2 | 16.9 | 20.3 | 43,600 | dominated |
| Cost-efficient screening strategies | | | | |  |  |  |
| FIT 50 | 50 | 80 | 2 | 35.0 | 39.5 | -500,200 | -12,700 |
| FIT 50 | 50 | 80 | 1.5 | 40.9 | 41.8 | -490,000 | 4,400 |
| FIT 50 | 45 | 79.5 | 1.5 | 48.8 | 46.5 | -404,700 | 18,400 |
| FIT 50 | 45 | 84 | 1.5 | 49.3 | 46.5 | -401,700 | 46,200 |
| FIT 50 | 45 | 80 | 1 | 58.6 | 48.8 | -280,600 | 53,800 |
| FIT 50 | 45 | 85 | 1 | 59.1 | 48.8 | -277,500 | 88,000 |
| FIT 75 | 40 | 85 | 1 | 58.8 | 50.6 | -94,600 | 99,500 |
| FIT 50 | 40 | 85 | 1 | 69.1 | 51.3 | -19,100 | 111,500 |

**Non-bleeding adenomas: 74% of large adenomas could not be detected by gFOBT and FIT[25]**

| **Screen test** | **Start age** | **Stop age** | **Interval** | **Col/year** | **QALY (years)** | **Costs ($)** | **ICER ($)** |
| --- | --- | --- | --- | --- | --- | --- | --- |
| Current screening strategy in Ontario | | | | |  |  |  |
| gFOBT | 50 | 74 | 2 | 15.1 | 16.8 | 202,900 | dominated |
| Cost-efficient screening strategies | | | | |  |  |  |
| FIT 50 | 55 | 70 | 1 | 33.4 | 30.9 | -61,700 | -2,000 |
| FIT 50 | 50 | 70 | 1 | 43.9 | 38.6 | -30,500 | 4,100 |
| FIT 50 | 50 | 75 | 1 | 46.4 | 39.4 | -20,400 | 12,400 |
| FIT 50 | 45 | 75 | 1 | 56.0 | 44.5 | 100,200 | 23,600 |
| FIT 50 | 45 | 80 | 1 | 57.0 | 44.7 | 113,500 | 73,000 |
| FIT 50 | 40 | 80 | 1 | 67.2 | 47.4 | 312,900 | 73,800 |
| FIT 50 | 40 | 85 | 1 | 67.6 | 47.4 | 321,200 | 417,000 |

**Rate of fatal complications: 1 per 28,000 colonoscopies (50% of base case value)[24]**

| **Screen test** | **Start age** | **Stop age** | **Interval** | **Col/year** | **QALY (years)** | **Costs ($)** | **ICER ($)** |
| --- | --- | --- | --- | --- | --- | --- | --- |
| Current screening strategy in Ontario | | | | |  |  |  |
| gFOBT | 50 | 74 | 2 | 16.9 | 20.5 | 221,000 | dominated |
| Cost-efficient screening strategies | | | | |  |  |  |
| FIT 50 | 55 | 74,5 | 1.5 | 31.6 | 34.1 | -354,200 | -10,400 |
| FIT 50 | 50 | 80 | 1.5 | 41.0 | 42.1 | -354,100 | 0 |
| FIT 50 | 50 | 80 | 1 | 49.4 | 44.4 | -325,500 | 12,500 |
| FIT 50 | 45 | 79.5 | 1.5 | 48.8 | 46.9 | -282,900 | 17,400 |
| FIT 50 | 45 | 80 | 1 | 58.6 | 49.3 | -195,500 | 35,600 |
| FIT 50 | 40 | 80 | 1 | 68.7 | 52.0 | 44,600 | 88,900 |
| FIT 50 | 40 | 85 | 1 | 69.1 | 52.1 | 48,300 | 126,400 |

**Rate of fatal complications: 1 per 7,000 colonoscopies (200% of base case value)[24]**

| **Screen test** | **Start age** | **Stop age** | **Interval** | **Col/year** | **QALY (years)** | **Costs ($)** | **ICER ($)** |
| --- | --- | --- | --- | --- | --- | --- | --- |
| Current screening strategy in Ontario | | | | |  |  |  |
| gFOBT | 50 | 74 | 2 | 16.9 | 20.1 | 220,800 | dominated |
| Cost-efficient screening strategies | | | | |  |  |  |
| FIT 50 | 55 | 74.5 | 1.5 | 31.6 | 33.4 | -354,400 | -10,600 |
| FIT 50 | 50 | 80 | 1.5 | 40.9 | 41.2 | -354,300 | 0 |
| FIT 50 | 50 | 80 | 1 | 49.4 | 43.2 | -325,900 | 14,100 |
| FIT 50 | 45 | 79.5 | 1.5 | 48.8 | 45.6 | -283,500 | 18,000 |
| FIT 50 | 45 | 80 | 1 | 58.6 | 47.7 | -196,100 | 41,400 |
| FIT 50 | 40 | 80 | 1 | 68.7 | 49.8 | 43,900 | 115,400 |
| FIT 50 | 40 | 85 | 1 | 69.0 | 49.8 | 47,600 | 137,100 |

**CRC relative survival: 25% improved survival, compared to base case values for all CRC stages**

| **Screen test** | **Start age** | **Stop age** | **Interval** | **Col/year** | **QALY (years)** | **Costs ($)** | **ICER ($)** |
| --- | --- | --- | --- | --- | --- | --- | --- |
| Current screening strategy in Ontario | | | | |  |  |  |
| gFOBT | 50 | 74 | 2 | 16.9 | 16.1 | -13,800 | dominated |
| Cost-efficient screening strategies | | | | |  |  |  |
| FIT 50 | 50 | 80 | 1.5 | 40.9 | 33.6 | -785,000 | -23,400 |
| FIT 50 | 50 | 80 | 1 | 49.4 | 35.4 | -776,200 | 4,800 |
| FIT 50 | 45 | 79.5 | 1.5 | 48.8 | 37.0 | -766,700 | 6,200 |
| FIT 50 | 45 | 80 | 1 | 58.6 | 39.1 | -708,400 | 27,800 |
| FIT 75 | 40 | 80 | 1 | 68.7 | 40.9 | -480,800 | 125,400 |
| FIT 50 | 40 | 85 | 1 | 69.0 | 40.9 | -477,800 | 156,200 |

**Increased cost of FIT: CAN$43.87 per test (based on reimbursement rate in the US Medicare program)[26]**

| **Screen test** | **Start age** | **Stop age** | **Interval** | **Col/year** | **QALY (years)** | **Costs ($)** | **ICER ($)** |
| --- | --- | --- | --- | --- | --- | --- | --- |
| Current screening strategy in Ontario | | | | |  |  |  |
| gFOBT | 50 | 74 | 2 | 16.9 | 20.3 | 220,900 | dominated |
| Cost-efficient screening strategies | | | | |  |  |  |
| FIT 50 | 55 | 74,5 | 1.5 | 31.6 | 33.8 | -295,000 | -8,700 |
| FIT 50 | 45 | 79.5 | 1.5 | 48.8 | 46.5 | -179,900 | 19,800 |
| FIT 50 | 45 | 80 | 1 | 58.6 | 48.8 | -65,800 | 49,300 |
| FIT 50 | 40 | 80 | 1 | 68.7 | 51.3 | 203,000 | 106,500 |
| FIT 50 | 40 | 85 | 1 | 69.1 | 51.3 | 207,900 | 177,900 |

**Decreased colonoscopy costs, 50% of base case value**

| **Screen test** | **Start age** | **Stop age** | **Interval** | **Col/year** | **QALY (years)** | **Costs ($)** | **ICER ($)** |
| --- | --- | --- | --- | --- | --- | --- | --- |
| Current screening strategy in Ontario | | | | |  |  |  |
| gFOBT | 50 | 75* | 2 | 16.9 | 20.3 | 45,000 | dominated |
| Cost-efficient screening strategies | | | | |  |  |  |
| FIT 50 | 45 | 80 | 1 | 58.6 | 48.8 | -849,200 | -10,800 |
| FIT 50 | 45 | 85 | 1 | 59.1 | 48.8 | -848,200 | 27,600 |
| FIT 50 | 40 | 85 | 1 | 69.1 | 51.3 | -761,600 | 34,400 |

**Increased colonoscopy costs, 200% of base case value**

| **Screen test** | **Start age** | **Stop age** | **Interval** | **Col/year** | **QALY (years)** | **Costs ($)** | **ICER ($)** |
| --- | --- | --- | --- | --- | --- | --- | --- |
| Current screening strategy in Ontario | | | | |  |  |  |
| gFOBT | 50 | 75* | 2 | 16.9 | 20.3 | 572,800 | dominated |
| Cost-efficient screening strategies | | | | |  |  |  |
| FIT 50 | 60 | 70 | 2 | 18.0 | 22.7 | 129,100 | 5,700 |
| FIT 50 | 55 | 70 | 3 | 19.9 | 27.0 | 155,000 | 6,000 |
| FIT 50 | 55 | 75* | 3 | 21.2 | 27.9 | 167,500 | 14,000 |
| FIT 50 | 55 | 70* | 2 | 24.2 | 30.0 | 197,000 | 14,100 |
| FIT 50 | 55 | 75 | 2 | 27.4 | 31.8 | 223,400 | 14,800 |
| FIT 100 | 50 | 75* | 1.5 | 27.1 | 37.4 | 324,900 | 18,100 |
| FIT 150 | 50 | 75 | 1 | 29.1 | 40.0 | 383,400 | 22,500 |
| FIT 150 | 45 | 80 | 1 | 36.2 | 45.5 | 645,500 | 47,500 |
| FIT 100 | 45 | 80 | 1 | 42.7 | 46.8 | 758,000 | 91,100 |
| FIT 150 | 40 | 80 | 1 | 42.5 | 49.0 | 975,700 | 97,500 |
| FIT 100 | 40 | 80 | 1 | 50.2 | 49.9 | 1,144,300 | 183,200 |
| FIT 100 | 40 | 85 | 1 | 50.8 | 50.0 | 1,163,800 | 268,000 |
| FIT 75 | 40 | 85 | 1 | 58.8 | 50.6 | 1,384,200 | 347,400 |
| FIT 50 | 40 | 85 | 1 | 69.1 | 51.3 | 1,667,200 | 417,700 |

**Decreased CRC treatment costs, 50% of base case value**

| **Screen test** | **Start age** | **Stop age** | **Interval** | **Col/year** | **QALY (years)** | **Costs ($)** | **ICER ($)** |
| --- | --- | --- | --- | --- | --- | --- | --- |
| Current screening strategy in Ontario | | | | |  |  |  |
| gFOBT | 50 | 75* | 2 | 16.9 | 20.3 | 414,600 | dominated |
| Cost-efficient screening strategies | | | | |  |  |  |
| FIT 50 | 55 | 70 | 3 | 19.9 | 27.0 | 165,400 | 6,100 |
| FIT 50 | 55 | 75* | 3 | 21.2 | 27.9 | 175,500 | 11,400 |
| FIT 50 | 50 | 75* | 2 | 26.8 | 34.4 | 250,600 | 11,600 |
| FIT 50 | 45 | 75 | 2 | 41.0 | 43.7 | 467,200 | 31,500 |
| FIT 50 | 45 | 80* | 2 | 41.9 | 44.0 | 479,400 | 42,700 |
| FIT 50 | 45 | 75 | 1.5 | 47.8 | 46.2 | 576,800 | 44,500 |
| FIT 50 | 45 | 80* | 1.5 | 48.8 | 46.5 | 590,500 | 54,800 |
| FIT 50 | 45 | 80 | 1 | 58.6 | 48.8 | 785,400 | 84,200 |
| FIT 100 | 40 | 80 | 1 | 50.2 | 49.9 | 883,600 | 85,900 |
| FIT 75 | 40 | 80 | 1 | 58.3 | 50.6 | 979,000 | 139,300 |
| FIT 50 | 40 | 80 | 1 | 68.7 | 51.3 | 1,100,700 | 174,900 |
| FIT 50 | 40 | 85 | 1 | 69.1 | 51.3 | 1,107,600 | 247,700 |

**Increased CRC treatment costs, 200% of base case value**

| **Screen test** | **Start age** | **Stop age** | **Interval** | **Col/year** | **QALY (years)** | **Costs ($)** | **ICER ($)** |
| --- | --- | --- | --- | --- | --- | --- | --- |
| Current screening strategy in Ontario | | | | |  |  |  |
| gFOBT | 50 | 75* | 2 | 16.9 | 20.3 | -166,400 | dominated |
| Cost-efficient screening strategies | | | | |  |  |  |
| FIT 50 | 45 | 85 | 1 | 59.1 | 48.8 | -2,160,000 | -44,300 |
| FIT 50 | 40 | 85 | 1 | 69.1 | 51.3 | -2,071,200 | 35,300 |

Col/year: colonoscopies per year; QALY: quality adjusted life years; ICER: incremental cost-effectiveness ratio.

The number of colonoscopies per year are undiscounted.

Costs (expressed in 2013 Canadian dollars) and QALYs are discounted by 3% per year.

* Stop age of screening is not necessarily the age of last screening. The last age of screening depends on start age and interval and is the latest age that can be acquired with that start age and interval that still is below the stop age of screening. For example, screening every 1.5 years from age 55 results in a final screening to be performed at the age of 74.5 years.
